# Modeling angiogenesis under Robin boundary conditions

**DOI:** 10.1002/qub2.70009

**Published:** 2025-06-13

**Authors:** Pablo Álvarez‐Caudevilla, Cristina Brändle, Elena Encinas

**Affiliations:** ^1^ Departamento de Matemáticas Universidad Carlos III de Madrid Leganés Madrid Spain

**Keywords:** angiogenesis, Keller–Segel, Robin boundary condition

## Abstract

In this study, we show an example of a numerical model based on the Keller–Segel system of equations to simulate angiogenesis in response to chemotaxis under Robin boundary conditions, which represent the presence of flux at the tumor. Different parameters of the model are modified to identify key biological factors relevant to the behavior of angiogenesis. The results show that in the presence of a stronger flux, angiogenesis occurs later owing to the chemical flux that creates a more uniform and homogeneous matrix, decreasing the pronunciation of the gradient and reducing the potential of chemotaxis.

## 1 INTRODUCTION

In 1971, Dr. Folkman stated: “The growth of solid neoplasms is always accompanied by neovascularization” [1]. That concept suggested that tumors are firmly dependent on the growth of a new vasculature to supply nutrients and oxygen. The physiological process of forming a new vasculature from preexisting blood vessels is denominated angiogenesis, and successful therapy in cancer involves dealing with blocking or reducing the formation of this blood supply.

Thus, angiogenesis, the process by which new blood vessels develop from an existing vasculature, is a natural and healthy process that plays an important role in reproduction and healing. It provides oxygen and nutrients to tissues and organs through the blood. Furthermore, it plays a key role not only in wound healing and the development of embryos but also in tumor growth [2], when cells start to compete for the limited number of resources in the environment. This hypoxic condition triggers the secretion of chemoattractants by the cancerous cells, called tumor angiogenic factor (TAF). These chemicals diffuse along the extracellular matrix, creating a concentration differential between the parent vessel and the tumor. The endothelial cells (ECs) respond to the presence of these angiogenic factors by moving and reproducing to generate a new vascular net attracted by the TAF, a process known as chemotaxis. Along with these chemical signals, the extracellular matrix provides cues to conduct the cellular motion [3]. Hence, the growth of the tumor, the location of the tumor about existing blood vessels, the protein makeup, and the permeability of the surrounding extracellular matrix are all factors that influence the tumor’s ability to generate new blood vessels.

Biology alone may not be enough to understand the complexity of angiogenesis. For this reason, mathematical models and computational simulations are employed. In 1970, Keller and Segel [4] proposed a mathematical model based on a system of two diffusion equations to study angiogenesis arising from chemotaxis, which has become the classical model to describe the process of chemotaxis. The diffusion of the chemoattractant and the movement of cells following a chemical cue were stated in just two related differential equations. This chemical gradient modified the direction and speed of migration of these cells, always considering biased random walk. The chemotactic response was considered analogous to Brownian motion, whereas changes in the paths of cell migration were determined by a chemical gradient. They explained that in the long term, a macroscopic flux could represent these microscopic processes when a high number of cells was averaged over. Nowadays, the model shown in the study by Keller and Segel [4] has demonstrated its potential to accurately predict the behavior of bacteria under certain environments, and it has been utilized to test the value of these predictions in several experiments.

Building upon this canonical model, many studies have been developed on mathematical models of angiogenesis incorporating chemotactic effects, analyzed from both mathematical and numerical viewpoints; see the study by Markowich and Oelz [5] and its references. However, these studies have been analyzed assuming homogeneous Neumann (no‐flux) boundary conditions. From a theoretical point of view, most of them have analyzed models consisting of two differential equations with Neumann boundary conditions, obtaining existence and multiplicity of solutions results using classical variational methods such as those performed by Chen and Zhong [6] or Fujie and Jiang [7] applying the mountain pass lemma and minimizing principles. A more experimental approach was performed, for example, by Anderson and Chaplain [8] or Mantzaris et al. [3]. They assume that the TAF concentration has reached a steady state, which provides the initial conditions for the TAF concentration profile, and, as ECs migrate through the extracellular matrix in response to this steady‐state gradient, there is just some uptake and binding of TAF by the cells.

The novelty of this paper, and the main difference from the study by Anderson and Chaplain [8], is that we provide numerical simulations for an angiogenic model with diffusion on both variables and chemotactic effects under Robin boundary conditions on TAF. Considering a general model of the form

(1) $$\begin{array}{rl} & \frac{\partial u}{\partial t}=\nabla \cdot \left(D_{u}\nabla u-G(v)u\nabla v\right)+f(u,v),\\ & \frac{\partial v}{\partial t}=D_{v}\Delta v+g(u,v), \end{array}$$

in a bounded domain Ω, with $D_{u},D_{v}$ standing for the diffusion coefficients, as mentioned above, most of the previous studies have focused on the analysis of such problems under homogeneous boundary conditions. However, in recent years there has been substantial interest in related systems with non‐Neumann boundary conditions in the second component, v; see, for instance, the survey in Ref. [9] or [10]. In the former, the authors assume Robin boundary conditions for the TAF concentration. There, the existence of solutions to the associated elliptic system was obtained using bifurcation analysis as well as comparison methods. Additionally, assuming the function *f*(*u, v*) of Equation (1) in logistic form, one can check the studies by Lankeit [11], Braukhoff and Lankeit [12], and Braukhoff and Tang [13], where, for a chemotaxis‐Navier–Stokes system with nonhomogeneous boundary conditions, the global existence of weak solutions was shown. Moreover, based on the analysis delivered in the study by Braukhoff [14], it is possible to prove such a global existence in 2D and 3D, after transforming the problem into a homogeneous Neumann boundary problem, for a system of the type (Equation 1). As shown in those references, the logistic term leads to interesting new effects in chemotaxis systems, helping in some cases to perform the analysis of these systems. However, because the main purpose of this study is to analyze the effect of Robin boundary condition through numerical simulations, we take *f*(*u, v*) = 0 and *g*(*u, v*) = −*γuv* in Equation (1) and do not focus on adding extra terms, such as logistic terms, into the equation.

As a novelty, we propose a time‐dependent model in which not only the ECs diffuse through the tissue but also the TAF, constantly generated by the tumor, which is located at the boundary, differently from most of the previous studies [8], in the analysis of angiogenesis with chemotactic effects. The experiments were conducted over a fixed period of time, without reaching the steady state. This approach was determined to assess the initial and intermediate dynamics of angiogenesis, phases more biologically relevant for identifying potential medical treatments. Hence, we provide a quantitative analysis of a problem emerging from angiogenesis with chemotaxis and flux at the boundary of a tumor for a fixed period of time, identifying and quantifying the essential parameters that play a key role in the system by modifying certain factors. Thus, some intrinsic information might be ascertained that could help reduce the chances of connection between the vessel and the tumor, as well as set a basis for future and more detailed mathematical models. We focus on the migration of ECs, delimiting the internal layer of blood vessels through a given space, following a chemotactic gradient synthesized by the tumor. Notably, even a simple change in the boundary conditions causes differences in the model’s solution behavior, which has significant implications for future mathematical and numerical analysis of these types of problems.

We will consider the effect of chemotaxis on angiogenesis and neglect the effect of haptotaxis. In general, we observe that the results obtained considering haptotaxis are quite equivalent to the ones omitting it (see, for example, Ref. [15]), so that the problem can be simplified by focusing only on the chemotaxis field.

Considering the influence of the parameters in the system, we point out that, in line with the findings of Mantzaris et al. [3] and Pamuk et al. [16], the biologically oriented model they analyzed contains substantially more parameters, equations, and therefore variables than our current model. However, it has been proved that using methods based on parameter space compression, for example, the so‐called manifold boundary approximation method due to Transtrum et al. [17], the models might be reduced to a more manageable model providing the same conclusions. In such an analysis, the fluctuation of the parameters shows that some of the parameters, despite their importance from a biological point of view, can be neglected because their effect on the conclusions is minimal. Those kinds of methods show the robustness of the models and the effectiveness of simpler models. Basically, through those methods, it is possible to identify, using some data obtained for the problem at hand, the parameters with less influence in the model and combine them such that the model becomes much simpler.

Consequently, supported by numerical results, the goal is to identify and quantify the essential parameters that play a key role in the system by modifying certain factors. Thus, some intrinsic information might be ascertained to help reduce the chances of connection between the vessel and the tumor, as well as set a basis for future and more detailed mathematical models. We also note that the identification of biological parameters is key to directing new treatments and is used as a double check to add accuracy to biological functions. The theoretical variation of the parameters of this model deduces some information to predict their effects on the behavior of angiogenesis.

## 2 RESULTS

### 2.1 A mathematical model for angiogenesis

**FIGURE 1:**
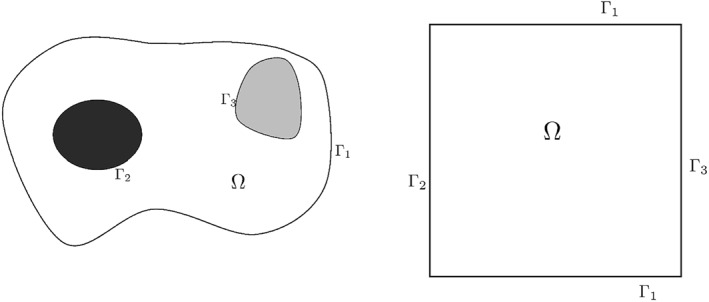
Configuration of the domain.

We present a mathematical model that aims to describe the evolution and migration process of ECs following the chemical gradient just formed by the release of TAF by the tumor. Hence, we consider two concentrations that correspond to the ECs, given by *u*, and the TAF concentration generated by the tumor, represented by *v*, living together during a certain period (0, *T*) in a region Ω *c R* ^2^, which is used to model the extracellular matrix and is assumed to be bounded and connected. Following Ref. [10], we split the boundary ∂Ω into three pieces, Γ_1_ ∪ Γ_2_ ∪ Γ_3_, with Γ*_i_* ∩ Γ*_j_* = ∅, for i≠j, so that both the tumor and the primary blood vessel are inside Γ_1_, Γ_2_ stands for the boundary of the tumor, and Γ_3_ stands for the boundary of the blood vessel; see Figure 1 for a possible example of such a configuration and its numerical simplification.

Of course, the conversion of a three‐dimensional tissue structure into a two‐dimensional model lattice with defined boundaries exposes some limitations in obtaining conclusive results. A three‐dimensional model will be closer to mimicking what happens in biology and will give results more similar to cellular responses from in vivo studies, as it will exhibit the complex interaction of cells with the matrix. However, mathematically analyzing a 2D model presents important conclusions to avoid geometric difficulties. We also observe that even in 2D, the shape of the tumor plays a more important role in the spread of tumoral cells and metastasis development than the analysis in 2D or 3D.

The system that describes the EC migration and the change in chemical concentration, TAF, over time, is given by the following equation:

(2) $$\begin{array}{rl} & \frac{\partial u}{\partial t}=\nabla \cdot \left(D_{u}\nabla u-\frac{\chi }{1+\alpha v}u\nabla v\right),\\ & \frac{\partial v}{\partial t}=D_{v}\Delta v-\gamma uv, \end{array}\;\text{in}\;\Omega \times (0,T),$$

where *D*_*u*_, *D*_*v*_ > 0 stand for the diffusion coefficients. The term *χ*(*v*) := *χ*(1 + *αv*)^−1^ is the chemotaxis factor, where *χ* and *α* are two positive constants, representing the maximum chemotactic response and the severity of desensitization, respectively (check Ref. [8] for further details). The parameter *γ* is identified as the interaction coefficient between ECs and TAF. Note that the existence of solutions for any time and the convergence to the steady‐state solutions were studied by Knosalla [18] and Knosalla and Nadzieja [19] for a similar problem, however, with different boundary conditions and in 1D.

Equation (2) states that the change of cell density over time is determined by the divergence of the total cell flux, which is composed of the random motion of cells and the chemotactic factors. In particular, we consider that EC is affected by a chemotaxis term, so that ECs move toward higher concentrations of TAF, depending on such a chemotaxis factor. Moreover, we assume that the change in the chemical concentration over time (Equation 2) is given by the diffusion of the chemical along the domain, together with a proportional term of competition with the ECs, such that this later term couples the two equations.

Together with Equation (2), we consider some boundary conditions on ∂Ω × (0, *T*). The simplest type of boundary conditions are no‐flux conditions for the EC density and Dirichlet boundary conditions for the chemicals; see, for instance, Refs. [3, 8]. However, in our model, as already mentioned, we assume that the tumor is generating TAF. A growth rate in the equation (an additive term of the form +βv in Equation (2); see the study by Markowich and Oelz [5]) supposes that the TAF is originated inside Ω, which is not realistic. So, we have introduced the growth rate on the boundary condition; see the study by Delgado and Suárez [10] for further details mathematically justifying such a choice. This change is supported by the assumption that tumors generally present a leaky structure [20] that can be simulated by Robin conditions at their border. Hence, we have no‐flux boundary conditions for both variables. However, we additionally introduce a flux of the chemical TAF in the tumor by implementing the Robin condition with a positive coefficient μ on the boundary of the tumor, which represents the amount of TAF that the tumor is generating. Hence, we assume, for all t,

(3) $$\begin{array}{cc} & \frac{\partial u}{\partial n}=0,\text{on}\;\Gamma_{1}\cup \Gamma_{2}\cup \Gamma_{3},\\ & \frac{\partial v}{\partial n}-\mu v=0,\text{on}\;\Gamma_{2},\\ & \frac{\partial v}{\partial n}=0,\text{on}\;\Gamma_{1}\cup \Gamma_{3}, \end{array}$$

where ***n*** denotes the outward unit normal to the boundary. Finally, we assume initial data, which are nonnegative and regular.

### 2.2 Computational results—Examples

In this subsection, we show the results obtained in this paper by solving the discretized version of Equation (2) under the boundary conditions of Equation (3) and initial data by considering different values for the parameters involved in the problem.

The approach performed here considers that the system holds on a square spatial domain with the parent vessel located along one edge and the tumor located on the opposite edge. Hence, we consider for the spatial domain a square of side 1, so that Ω=(0,1)×(0,1). The tumor and the parent vessel are located at the boundary of Ω, so that $\Gamma_{1}=\left(0,1\right)\times \left(\left\{0\right\}\cup \left\{1\right\}\right)$, $\Gamma_{2}=\left\{0\right\}\times \left(0,1\right)$, and $\Gamma_{3}=\left\{1\right\}\times \left(0,1\right)$; see Figure 1. The time interval is chosen to be (0,2). To compute numerical solutions, we use the scheme introduced by Saito [21] to discretize the equation that describes the behavior of u and finite differences for v. We omit here the details, because they are out of the scope of this paper, and refer the interested reader to these articles.

**FIGURE 2:**
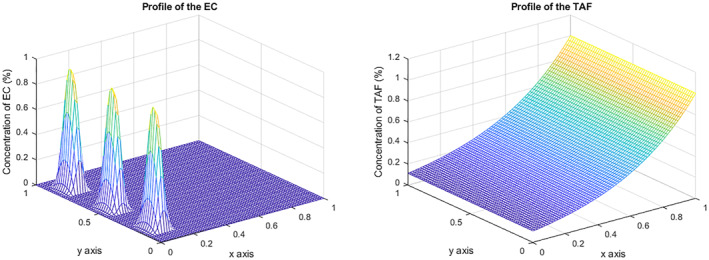
Initial EC and TAF concentration. EC, endothelial cell; TAF, tumor angiogenic factor.

According to this spatial configuration, we fix the initial distribution of ECs and TAF as follows: we assume that the tumor, *v*, is represented by a long row, and for the parent vessels, *u*, and inspired by Anderson and Chaplain [8], we consider a three‐peak initial configuration

$$\begin{array}{cc} & u(x,y,0)=\max\left\{0,{\text{e}}^{-y^{2}⁄0.001}\;\sin(6\pi x)\right\},\\ & v(x,y,0)={\text{e}}^{-(1-y)⁄0.45}, \end{array}$$

choosing (1−y) to ensure the compatibility of the boundary data; see the study by Anderson and Chaplain [8]. Observe that on the opposite side, theoretically, we do not have either Dirichlet homogeneous or Neumann homogeneous data; however, from a numerical point of view, both conditions are satisfied; see Figure 2.

We also fix the initial values of the parameters appearing in the system of Equations (2) and (3) as follows:

(4) $$D_{u}=0.0035,D_{v}=0.01,\chi =1,\alpha =0.6,\gamma =0.1,\mu =0.5.$$

We will then vary the values of χ,μ, and $D_{v}$ to get a precise map of the influence of each of them on the behavior of the system. The variables, controlling the saturation effect of TAF receptors of ECs, and γ, determining the consumption speed of TAF by ECs, were excluded from this study. These parameters represent intrinsic properties of ECs’ response to TAF and are defined by established biological principles. Instead, this analysis focuses on exploring the extrinsic factors of angiogenesis, those determined by the environment or other external influences.

Each parameter was changed independently, whereas the others were kept constant at their benchmarking values, to capture the explicit implication of the altered variable. Hence, unless otherwise indicated, the values of the parameters used for the simulations presented in this paper were given by Equation (4). Regarding the choice of parameters, our computations are naturally scaled in millimeters (mm) for length and in units of time (0<t<T).

**FIGURE 3:**
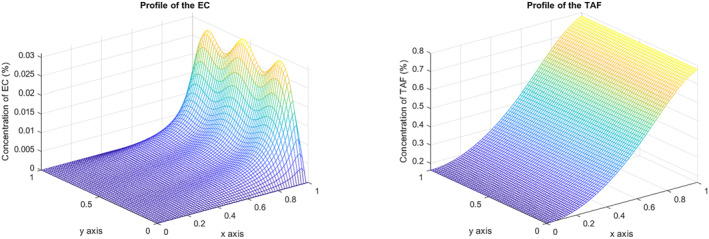
EC and TAF concentration at *T* = 2. EC, endothelial cell; TAF, tumor angiogenic factor.

Given the parameters in Equation (4), the three vessel tips reach the other side of the domain for a fixed time T and a concentration at this location of 0.03%; the maximum concentration of TAF at this point is 0.8%; see Figure 3.

**FIGURE 4:**
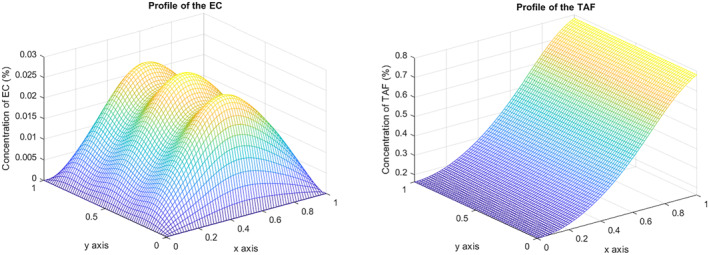
EC and TAF concentration at *T* = 2 when *χ* is halved, *χ* = 0.5. EC, endothelial cell; TAF, tumor angiogenic factor.

Regarding identifying critical model parameters, interesting results can be seen when the chemotactic sensitivity, χ, is halved. This points out that ECs’ influence on the chemical gradient is reduced. In this sense, Figure 4 shows how they do not reach the tumor for time T=2. On the other hand, the final concentration compared with the reference does not vary. The parameter χ characterizes the cell chemotactic response. The higher its value, the stronger chemotaxis’ influence on the vasculature’s growth. Hence, the dependence of ECs on TAF would cause cells to diffuse more slowly across the domain.

**FIGURE 5:**
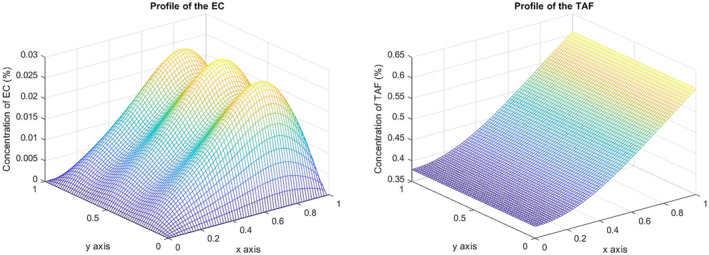
EC and TAF concentration at *T* = 2 when *D*_v_ is increased, *D*_v_ = 0.1. EC, endothelial cell; TAF, tumor angiogenic factor.

Next, we analyze the impact of the diffusion coefficient for the tumor chemical, $D_{v}$. This parameter was increased by a factor of 10. Figure 5 represents the solution when the diffusion term is $D_{v}=0.1$.

This change delayed the movement of the cells, even though the final concentration was unchanged. Furthermore, there is a notable change in the shape of the three tips adopted at the end of the simulation. Similarly, the TAF profile shows a decrease in its curvature due to the increase in this diffusion coefficient, leading to a more homogeneous chemical distribution along the domain. The $D_{v}$ diffusion parameter, reflecting the chemical random mobility, has a crucial role in shaping the chemotactic gradient. Larger values of $D_{v}$ increase the TAF diffusion in the domain and lead to a smoother chemical gradient, thereby diminishing ECs’ attraction. From a biological perspective, enhancing molecular diffusion in the medium may assist as an approach to avoid neovascularization. Additionally, the physical characteristics of the tissue between the tumor and the parent vessel hold prognostic significance. For example, a tissue microenvironment containing cartilage, bone, or dense fiber tissues will make chemical diffusion difficult, resulting in a steeper chemical gradient than a medium lacking such structural barriers, which enables the chemical to diffuse freely.

**FIGURE 6:**
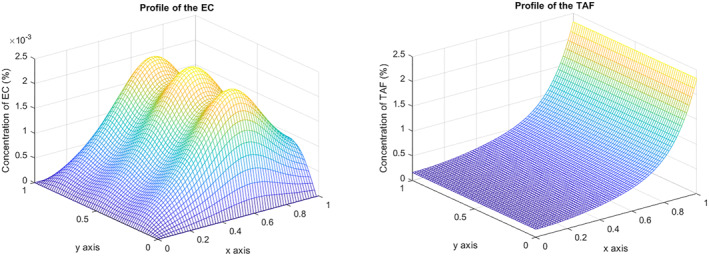
EC and TAF concentration at *T* = 2 when *μ* is increased, *μ* = 5. EC, endothelial cell; TAF, tumor angiogenic factor.

**FIGURE 7:**
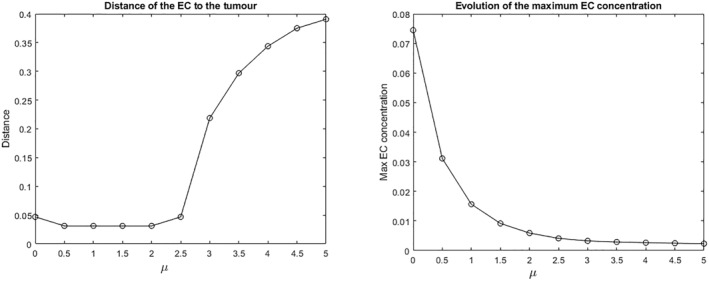
Evolution of the maximum EC concentration values of μ=0,0.5,…,5. EC, endothelial cell.

The main novelty of this study lies in capturing the importance of chemical flux at the tumor interface. Hence, we deal with the boundary condition and study the effect of increasing the amount of TAF the tumor generates. Our first simulation consists of increasing the parameter μ to 5. It can be observed that angiogenesis does not take place, and the cells present a quite low concentration by the end of the experiment (10^−3^%). The shape of the chemical gradient experiences a significant change and a strong increment in the concentration from 0.8% to 2.5% (Figure 6). In Figure 7, the maximum concentration of EC at the final time T=2 is shown for values of *μ* ranging from μ=0 (which corresponds to a Neumann boundary condition) to μ=5. Not only does the concentration of EC decrease with increasing μ but also the rate at which the EC moves decrease and, consequently, their distance from the tumor source increases.

This experiment confirmed that an increment in flux correlated with slower cell migration and reduced growth. This may be explained by the continuous diffusion of TAF across the domain, which progressively decreases the steepness of the gradient. Consequently, the chemotactic effect weakens due to the more homogeneous distribution of TAF, reducing the attraction of ECs and finally slowing their mobility.

## 3 CONCLUSIONS

Numerical simulations are carried out to reproduce the vasculature growth following a chemical gradient, depending on the consideration of flux at the tumor boundary throughout this paper. The technique that is used to follow the path of an endothelial mass of tissue is overcome by the discretization of a system of partial differential equations that governs the rate of change of cell density, depending on chemotaxis. Therefore, it is necessary to define the scope of the problem, and for that, a situation is considered where the location of the tumoral mass is on one side of a square domain and the parent vessel is on the opposite side. The results obtained in this analysis let us evaluate the specific processes involved in angiogenesis and how to correlate them to the parameters of the mathematical model.

It is found that a highly permeable tumor created a less aggressive chemical gradient, reducing the potential of angiogenesis arising from chemotaxis. In general, this conclusion allows associating a leaky structure in tumoral cells, which is more representative of the biological version, with a reduction in angiogenesis, as well as a detriment in the efficiency of the activity of this chemoattractant to the endothelium.

Moreover, by manipulating the different variables in the equations, we raise our comprehension of the relevance of each of them. For instance, our analysis shows some conclusions on the chemotactic sensitivity of ECs to TAF, given by the chemotaxis factor χ(v), standing for the tip cell sensitivity to TAF. We observe that the higher its value, the stronger the chemotaxis’ influence on the vasculature’s growth. Precisely, when χ is halved, the link between vessel and tumor is never created by the end of the simulations performed here. The TAF significance becomes weaker, as it could elevate to a larger concentration that is not going to trigger any trivial effect on cell chemotaxis. Consequently, reducing TAF dependence would cause cells to diffuse more slowly across the domain. This presents an opportunity to explore medicines that decrease cells’ chemotactic sensitivity.

Similarly, it has been shown how an increment in the diffusion of the chemical in the medium impedes the growth of blood vessels toward the tumor location. Indeed, the diffusion factor of the tumor chemical, $D_{v}$, characterizes the TAF random motility. This coefficient, when low, makes chemotaxis govern the equation; otherwise, diffusion triggers the lateral migration of the chemicals to one another along the *y*‐axis, and the step of the gradient is decreased, resulting in a delay in angiogenesis. In this context, increasing the chemical diffusion in the medium could be beneficial in preventing the formation of new blood vessels toward the tumor because there will be a point where the chemical gradient is not strong enough to lead to the growth of the vessels.

In conclusion, during the whole development of these numerical simulations, we analyze the biological events involved in angiogenesis through the effect that some parameters have on the proposed model.

## AUTHOR CONTRIBUTIONS

**Pablo Álvarez‐Caudevilla**: Writing—original draft; writing—review and editing. **Cristina Brändle**: Writing—original draft; writing—review and editing. **Elena Encinas**: Writing—original draft; writing—review and editing.

## CONFLICT OF INTEREST STATEMENT

The authors declare no conflicts of interest.

## ETHICS STATEMENT

This article does not contain any studies with human or animal materials performed by any of the authors.

## Data Availability

Data sharing is not applicable to this article as no new data were created or analyzed in this study.
